# Review on Chemical Constituents of *Schizonepeta* *tenuifolia* Briq. and Their Pharmacological Effects

**DOI:** 10.3390/molecules27165249

**Published:** 2022-08-17

**Authors:** Xueying Zhao, Mingwei Zhou

**Affiliations:** School Basic Medical Sciences, Heilongjiang University of Chinese Medicine, 24 Heping Road, Harbin 150040, China

**Keywords:** *Schizonepeta tenuifolia*, chemical constituents, volatile oil, pharmacological effects, H_2_O/alcohol extract

## Abstract

*Schizonepeta tenuifolia* Briq. is a famous Chinese traditional medicine with antipyretic, anti-inflammatory, analgesic and hemostatic effects. Many chemical components can be isolated and detected by using various analysis methods, including monoterpenes, sesquiterpenes, aldehydes, ketones, quinones, alcohols, phenols, carboxylic acids and esters, etc., in which volatile oil was considered to be the main chemical component. In this paper, the chemical constituents and their pharmacological effects were reviewed by summarizing the recent literature, revealing the relationship between them.

## 1. Introduction

*Schizonepetatenuifolia* (*ST*) Briq., also known in China as Jing Jie, belongs to the family Lamiaceae and is a perennial herbaceous plant and an herbal medicine that has been widely used for thousands of years in China, Japan and Korea. *ST* is a perennial plant with a firm stem, lignified base and many branches, and is 40–150 cm tall, subquadrilateral at the base, superficially an obtuse quadrilateral, lightly grooved and covered with white pubescence. It mostly grows near houses or in thickets where the elevation is generally no more than 2500 m. The dried aerial part of *ST* is applied clinically for diseases such as allergic skin disease, inflammatory skin disease, infectious skin disease and the common cold [1]. The main chemical constituent of *ST* is volatile oil, and other compounds, such as flavonoids, glycosides, etc., were detected. In addition, the volatile oil mainly contains terpenoids, aldehydes, ketones, quinones, alcohols, phenols, esters, carboxylic acids and alkenes. The chemical constituents of *ST* were extracted by steam distillation and ultrasonic and cold immersion methods, and analyzed by gas chromatography–mass spectrometry (GC-MS), high-performance liquid chromatography–mass spectrometry (HPLC-MS), nuclear magnetic resonance (NMR) spectra, etc. to confirm the structure of the extracted components [2,3,4]. Additionally, various extraction techniques such as solvent immersion, mechanical shaking and sonication were evaluated, and the greatest efficiency was observed with sonication extraction using petroleum ether [2]. Studies showed that different chemical constituents exhibit different pharmacological effects. This paper summarized the chemical constituents of *ST* and their pharmacological effects studied in recent years.

## 2. Chemical Constituents of *ST*

### 2.1. Volatile Oil

Volatile oils are considered to be the main constituents of *ST* that affect multiple pharmacological targets and provide clinical efficacy [3,4]. Pulegone is the indicator ingredient for the quality control of *ST* according to the stipulates of the *Chinese Pharmacopoeia* [1]. Zhu et al. analyzed the composition of the volatile oil from *ST* using GC-MS [5]. Table 1 lists the quantitative analysis results on volatile oil from *ST*, and each component was compared with *Menthae Haplocalycis Herba* (*MH*), as it is also from the Lamiaceae family. Among the constituents of volatile oil from *ST*, iso-menthone and pulegone were determined to be the two most abundant components. This paper summarizes monoterpenes, sesquiterpenes, aldehydes, ketones, quinones, alcohols, phenols, carboxylic acids and esters, and other compounds in order.

#### 2.1.1. Monoterpenes

**Figure 1:**
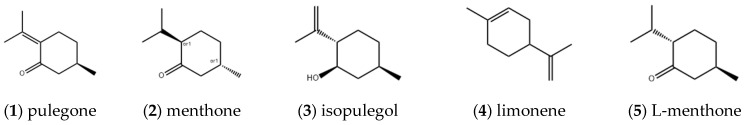

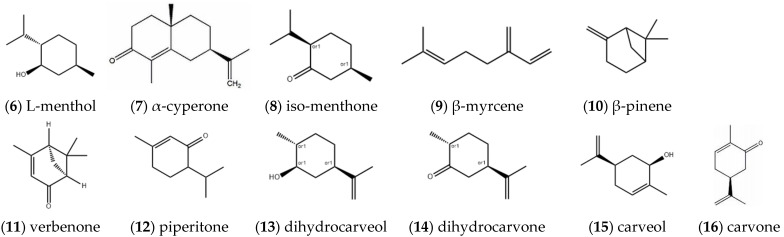
Structures of monoterpenes isolated from *ST*.

As shown in Figure 1, various monoterpenes were isolated from the volatile oil of *ST* [6,7,8,9,10]. Cai et al. used a supercritical CO_2_ extraction method to extract the volatile oil of *ST* and GC-MS combined technology to measure the chemical composition of the volatile oil [6,7,8,9,10]. The main monoterpenes in the volatile oil of *ST* were **pulegone** (1), **D-menthone** (2), **isopulegol** (3) and **limonene** (4). Three compounds, **L-menthone** (5), **iso-menthol** (6) and **α-cyperone** (7), were isolated by using gas chromatography-mass spectrometry to analyze the chemical components of volatile oil from *ST* spikes produced in Mengshan, Shandong, China [7]. Liu et al. obtained **iso-menthone** (8), **β-myrcene** (9), **β-pinene** (10), **verbenone** (11) and **piperitone** (12) by gas chromatography-mass spectrometry [8]. Qiu et al. used supercritical CO_2_ fluid extraction and steam distillation to extract the chemical components of volatile oil from the *Schizonepeta knifolia* spike, and obtained **menthofuran**, **perillyl alcohol**, **dihydroartemisia terpenol** (13), **dihydroartemisia terpenone** (14) and **carvatol** (15) [9]. Additionally, the compound **carvone** (16) was detected by GC-MS analysis from the volatile components of the *ST* ear [9]. Chun et al. also extracted and isolated **pulegone** (1), **menthone** (2) and other volatile components by using a simple and rapid gas chromatography/mass spectrometry (GC/MS) analysis method for the crude extract of *ST* [2]. A GC-MS-selected ion monitoring (SIM) detection method was developed for simultaneous determination of the monoterpenes, **menthone** (2), **pulegone** (1) and **limonene** (4), as the main bio-active and toxic constituents in the volatile oils of *ST* leaves and spikes at different harvesting times [3].

#### 2.1.2. Sesquiterpenes

**Figure 2:**
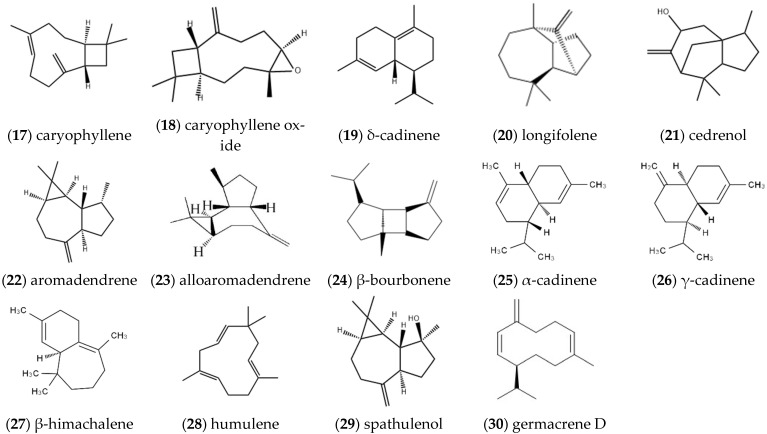
Structures of sesquiterpenes isolated from *ST*.

At present, more than 20 sesquiterpenes have been extracted and identified in *ST*. The main sesquiterpenes in the volatile oil of *ST* were isolated and elucidated as **caryophyllene** (17), **caryophyllene oxide** (18), **δ-cadinene** (19), **longifolene** (20), **cedrenol** (21) [9], **aromadendrene** (22), **betelaniene** (23), **β-bourbonene** (24), **α-cadinene** (25), **γ-cadinene** (26), **β-himachalene** (27) [6], **humulene** (28), **spathulenol** (29), etc. (Figure 2) [10]. Du et al. used GC-MS to analyze the chemical composition of the volatile oil of *ST* from different habitats and obtained the compound **germacrene D** (30); its structure is presented in Figure 2 [11].

#### 2.1.3. Aldehydes, Ketones and Quinones

**Figure 3:**
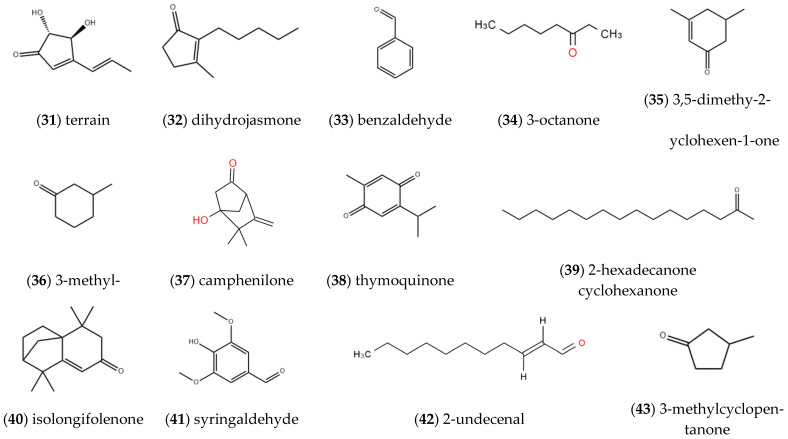
Structures of aldehydes, ketones and quinones isolated from *ST*.

As depicted in Figure 3, aldehydes, ketones and quinones in the volatile oil of *ST* mainly include **terrain** (31), **dihydrojasmone** (32) [6], **benzaldehyde** (33), **3-octanone** (34), **3,5-dimethyl-2-cyclohexene-1-one** (35), **3-methyl-1-cyclohexanone** (36), **camphenilone** (37), **thymoquinone** (38) [7], **2-hexadecanone** (39), **isolongifolenone** (40), **syringaldehyde** (41) and **2-undecenal** (42) [9]. Ye et al. studied the volatile oil components from the different parts of *ST* and obtained the chemical components of **3-methyl cyclopentanone** (43) [12].

#### 2.1.4. Alcohols and Phenolic Compounds

**Figure 4:**
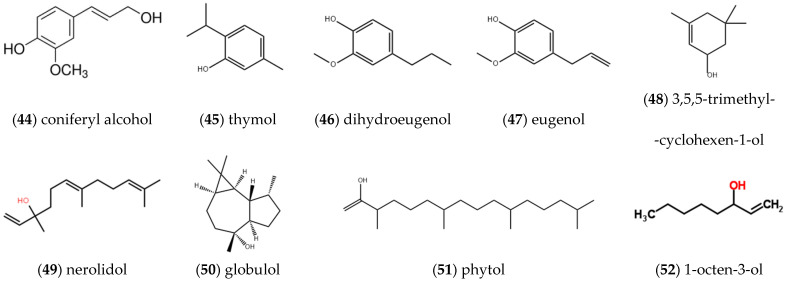
Structures of alcohols and phenolic compounds isolated from *ST*.

As displayed in Figure 4, a series of alcohols and phenolic compounds was extracted and detected in the volatile oil of *ST*, mainly including **coniferyl alcohol** (44), **thymol** (45), **dihydroeugenol** (46), **eugenol** (47) [7], **3,5,5-trimethyl-2-cyclohexen-1-ol** (48), **nerolidol** (49), **globulol** (50), **phytol** (51) [9] and **1-octene-3-ol** (52) [13].

#### 2.1.5. Carboxylic Acids and Esters

**Figure 5:**
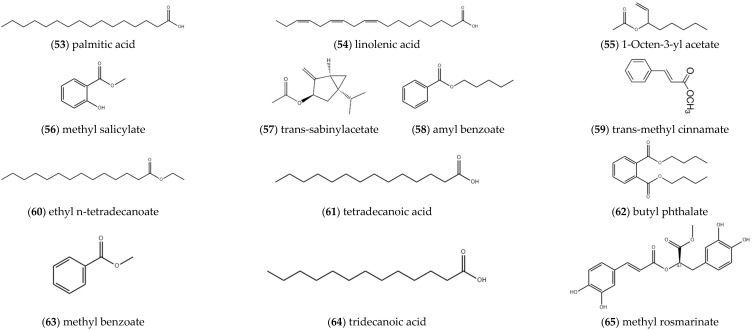
Structures of alcohols and phenolic compounds isolated from *ST*.

The researchers also obtained some carboxylic acid and ester compounds in the volatile oil of *ST*, and they are mainly **palmitic acid** (53), **linolenic acid** (54), **1-octen-3-yl acetate** (55) [11], **methyl salicylate** (56), **trans-sabinylacetate** (57), **amyl benzoate** (58), **trans-methyl cinnamate** (59), **ethyl n-tetradecanoate** (60) [7], **tetradecanoic acid** (61), **butyl phthalate** (62) [9] and **methyl benzoate** (63) (Figure 5) [10]. Yang et al. used GC-MS-DS technology to obtain the compound **tridecanoic acid** (64) [14]. Meng et al. used silica gel, Sephadex LH-20, ODS and semi-preparative HPLC columns to separate and purify the ethyl acetate portion of the 70% ethanol extract of *ST*, and identified the structure of the obtained compounds according to physicochemical properties and spectral data [15]. Rosmarinic acid **methyl ester** (65) was obtained (Figure 5).

#### 2.1.6. Alkenes and Alkanes

**Figure 6:**
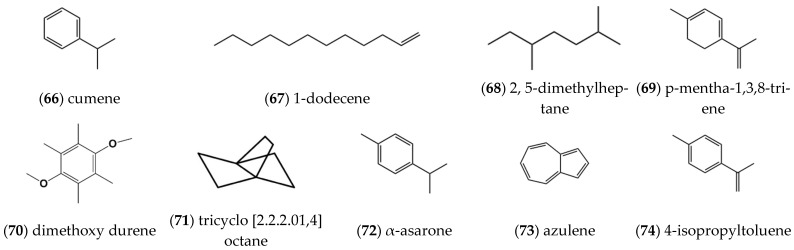
Structures of alkanes isolated from *ST*.

In addition to some common saturated n-alkanes such as **n-heneicosane**, **n-tetracosane**, **n-hexacosane**, **heptacosane**, **octacosane**, etc. [9], some other alkanes and olefin compounds were also extracted and isolated from the volatile oil of *ST*, and they included **kuprene** (66), **1-dodecene** (67), **2,5-dimethylheptane** (68) [7], **p-mentha-1,3,8-triene** (69), **dimethoxy durene** (70), **tricyclo [2.2.2.01,4] octane** (71), **α-asarone** (72), **azulene** (73) [8] and **4-isopropyltoluene** (74) [11] as depicted in Figure 6.

#### 2.1.7. Other Compounds

**Figure 7:**
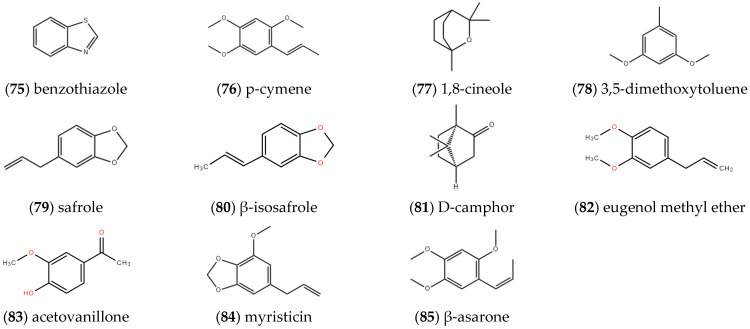
Structures of other compounds in volatile oil isolated from *ST*.

As shown in Figure 7, other compounds in the volatile oil of *ST* mainly include **benzothiazole** (75) [6], **p-cymene** (76), **1,8-cineole** (77) [8], **3,5-dimethoxyltoluene** (78), **safrole** (79) [11], **β-isosafrole** (80), **D-camphor** (81), **eugenol methyl ether** (82), **acetovanillone** (83), **myristicin** (84) and **β-asarone** (85) [9].

#### 2.1.8. Other Terpenoids

**Figure 8:**
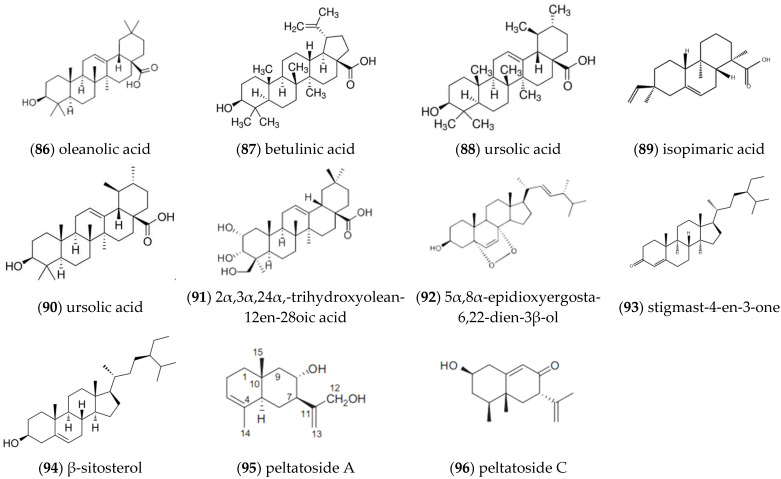
Structures of other terpenoids isolated from *ST*.

In addition to monoterpenes and sesquiterpenes isolated from volatile oil components, other terpenoids were also isolated and identified from *ST* (Figure 8). Meng et al. separated **oleanolic acid** (86), **betulinic acid** (87), **ursolic acid** (88), and **isopimaric acid** (89) from the ethyl acetate portion of the 70% ethanol extract of *ST* [14]. Lee et al. used column chromatography to separate MeOH extracts from the aboveground part of *ST*, and some other terpenoids were isolated including **ursolic acid** (90), **2α,3α,24α,-trihydroxyolean-12en-28oic acid** (91), **5α,8α-epidioxyergosta-6,22-diol-3β-ol** (92), **stigmast-4-en-3-one** (93) and **β-sitosterol** (94), as determined by a spectroscopic method [16]. Zhao et al. used various spectral techniques to analyze other terpenoids in *Schizonepeta* and obtained two compounds, **peltatoside A** (95) and **peltatoside C** (96) [17].

### 2.2. Flavonoids

**Figure 9:**
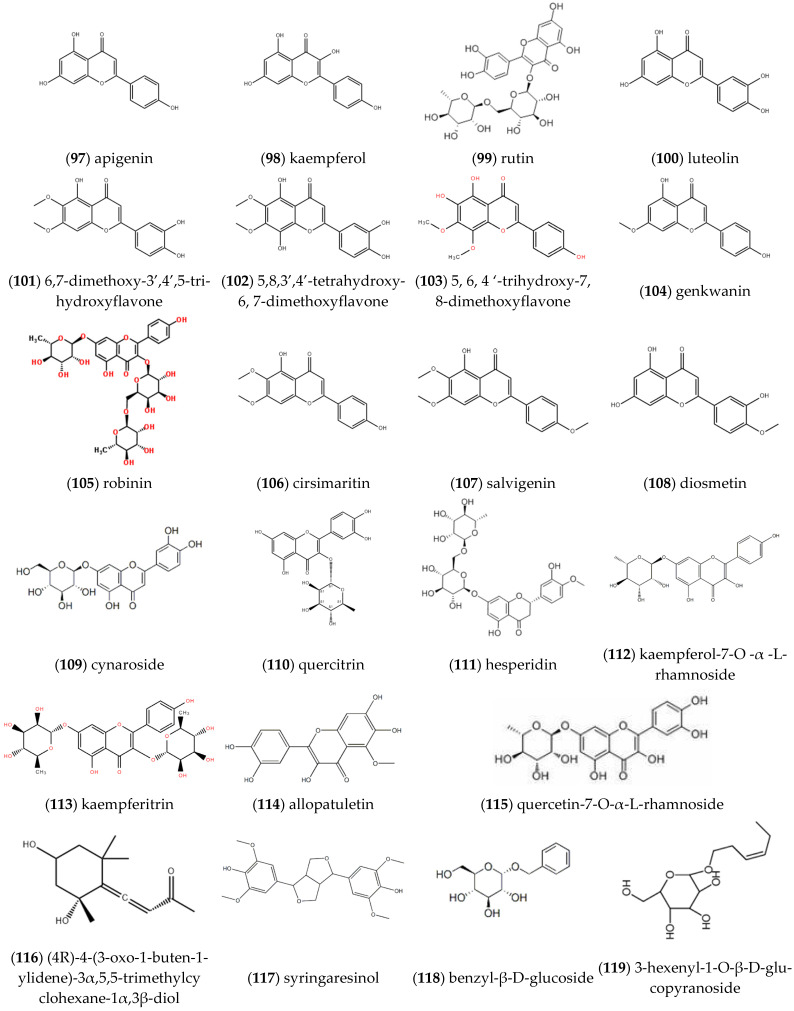

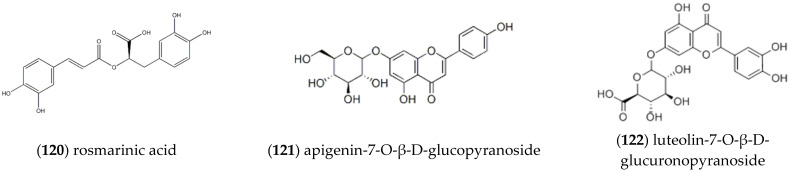
Structures of flavonoids isolated from *ST*.

As exhibited in Figure 9, lots of flavonoids were isolated and analyzed from *ST*, including **apigenin** (97), **kaempferol** (98), **rutin** (99), **luteolin** (100), **6,7-dimethoxy-3’,4’,5-trihydroxyflavone** (101), **5,8,3’,4’-tetrahydroxy-6,7-dimethoxyflavone** (102), **5,6,4’-trihydroxy-7,8-di-methoxyflavone** (103), **genkwanin (5,4′-dihydroxy-7-methoxy-flavone)** (104), **robinin** (105), **cirsimaritin** (106), **salvigenin** (107), etc. [18]. Moreover, Fan et al. detected the flavonoids from *ST* [19], and found that they were **diosmetin** (108), **cynaroside** (109), **quercitrin** (110), **hesperidin** (111) and other components. Furthermore, the content of flavonoids varied with the origin of the medicinal materials. Song et al. extracted and isolated **kaempferol-7-O-α-L-rhamnoside** (112), **kaempferitrin** (113), **allopatuletin** (114), **quercetin-7-O-α-L-rhamnoside** (115), **grasshopper ketone** **((4R)-4-(3-Oxo-1-buten-1-ylidene)-3α,5,5-trimethylcyclohexane-1α,3β-diol)** (116), **syringaresinol** (117) and **benzyl-β-D-glucoside** (118) from *ST* [20]. **3-hexenyl-1-O-β-D-glucopyranoside** (119), **rosmarinic acid** (120), **apigenin-7-O-β-D-glucopyranoside** (121) and **luteolin-7-O-β-D-glucuronopyranoside** (122) were also isolated and detected by Lee et al. [16].

Wen et al. determined the contents of caffeic acid, cynaroside, quercitrin, rosmarinic acid, luteolin, apigenin, diosmetin and pulegone in *ST* from 13 different origins in China by using HPLC, as listed in Table 2, and they found the contents of these compounds were different in different areas [21]. The fingerprint of *ST* in different areas was established to provide an experimental basis for the further comprehensive development and utilization of *ST*.

### 2.3. Other Compounds

**Figure 10:**
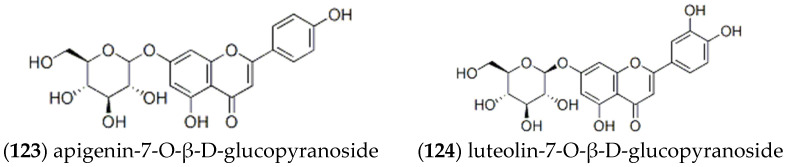

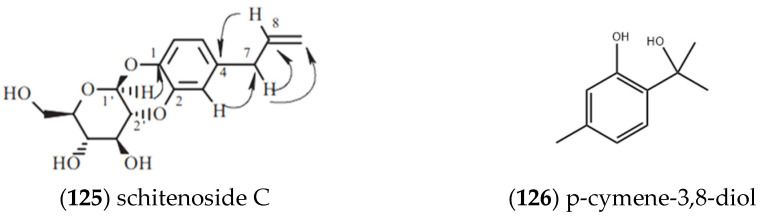
Structures of other compounds isolated from *ST*.

As displayed in Figure 10, Lee et al. used column chromatography to separate MeOH extract from the aboveground part of *ST* and isolated the phenolic compounds [16], which were **apigenin-7-O-β-D-glucopyranoside** (123) and **luteolin-7-O-β-D-glucopyranoside** (124). A new phenolic compound, **schitenoside C** (125), was also isolated from *ST* by repeated column chromatography [22]. Its structure was assigned by spectroscopic data interpretation. The compound **p-cymene-3,8-diol** (126) was for the first time isolated from *ST* by Lee et al. [16].

## 3. Pharmacological Effects

Modern pharmacological studies show that the extracts of ST have their respective pharmacological activities, such as antipyretic effects, antioxidant effects, hypoglycemic effects, anti-inflammatory effects, immunomodulatory effects, hemostatic effects, abirritation, antitumor effects, antibacterial effects, antiviral activity, etc.

### 3.1. Antipyretic Effect

Zhang et al. showed that nepetalactone (terpenoid compounds) could significantly reduce the body temperature of rats in a fever model, showing a significant antipyretic effect [23]. Cai et al. found that the antipyretic effect of the micropowder of *ST* could significantly inhibit the increase in body temperature in febrile rabbits [24]. The composite spray made from *ST* had significant antipyretic effects on influenza A and B virus models in mice [25]. *ST* showed an effective role in reducing the hemagglutinin titer in the lung tissue of influenza virus-infected mice in the prescription of the Yinqiao Decoction [26]. Bouididael et al. confirmed that nepetalactone could significantly inhibit the neurocentral system and enhance its antipyretic effect when combined with pentobarbital sodium [27].

### 3.2. Antioxidant Effect

The antioxidant activities and mechanism of the *ST* extract including phenolics, flavonoids and anthocyanin were explored by measuring free radical scavenging activity, viz, 1,1-diphenyl-2-picrylhydrazyl (DPPH), nitric oxide (NO), and deoxyribose oxidation levels. The results suggest that the methanol extract of *ST* can exert significant antioxidant activity via the inhibition of free radicals, iNOS and DNA oxidation [28]. Wang’s study showed that aqueous extracts of *ST*, as a natural inhibitor of oxidation and inflammation, displayed radical scavenging and reducing activity, as well as liposome protection activity [29].

**Figure 11:**
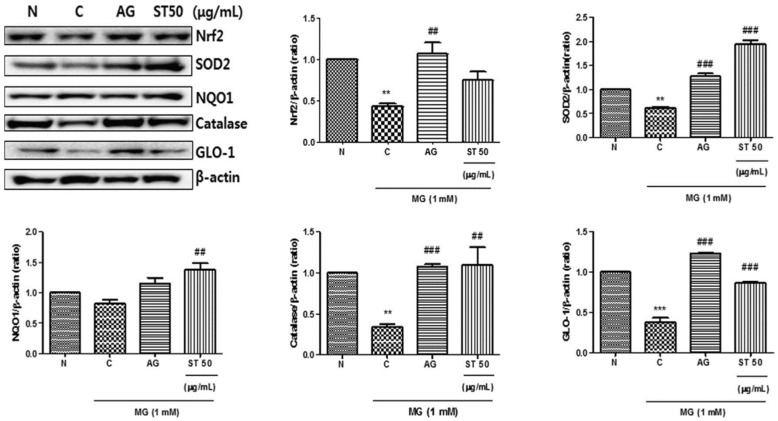
Effects of S. tenuifolia on the Nrf2/ARE pathway and GLO1 expression. The bar values are presented as mean ± SD of three independent experiments (** *p* < 0.01 and *** *p* < 0.001 vs. normal, ^##^ *p* < 0.01 and ^###^ *p* < 0.001 vs. control) (from Figure 5 in [31]).

Wen et al. found that the clearance rate of 1,1-diphenyl-2-trinitrophenylphenylhydrazine (DPPH) free radicals by the polysaccharide extract of *ST* was as high as 76.29%, and the activity of scavenging hydroxyl radical and superoxide anions was very high for both, indicating that the polysaccharide extract had good antioxidant activity [30]. DO et al. found that the extract of *ST* can improve the cytotoxicity and oxidative stress of mouse thylakoid cells by inhibiting the formation of AGEs and the crosslinking of AGE proteins [31], and they confirmed that *ST* can activate the Nrf2/ARE pathway. Their results suggest that *ST* has an inhibitory effect on MG-induced cytotoxicity by regulating the Nrf2/ARE pathway and reducing ROS production in renal cells, as shown in Figure 11.

Berner et al. found that the leaves of *ST* not only significantly increased the expression of the Nrf2 protein, but also significantly enhanced the expression of antioxidant enzymes [32]. Wang et al. investigated the antioxidant effects of vitex lignans, hesperidin and luteolin extracted from *ST* [29]. Qian et al. found that the total flavonoids in *ST* have scavenging ability on the hydroxyl radical (·OH), DPPH radical (DPPH·) and the superoxide anion radical (O_2_^−^·), especially showing much stronger scavenging ability on OH and O_2_^−^· [33].

### 3.3. Anti-Inflammatory Effect

**Figure 12:**
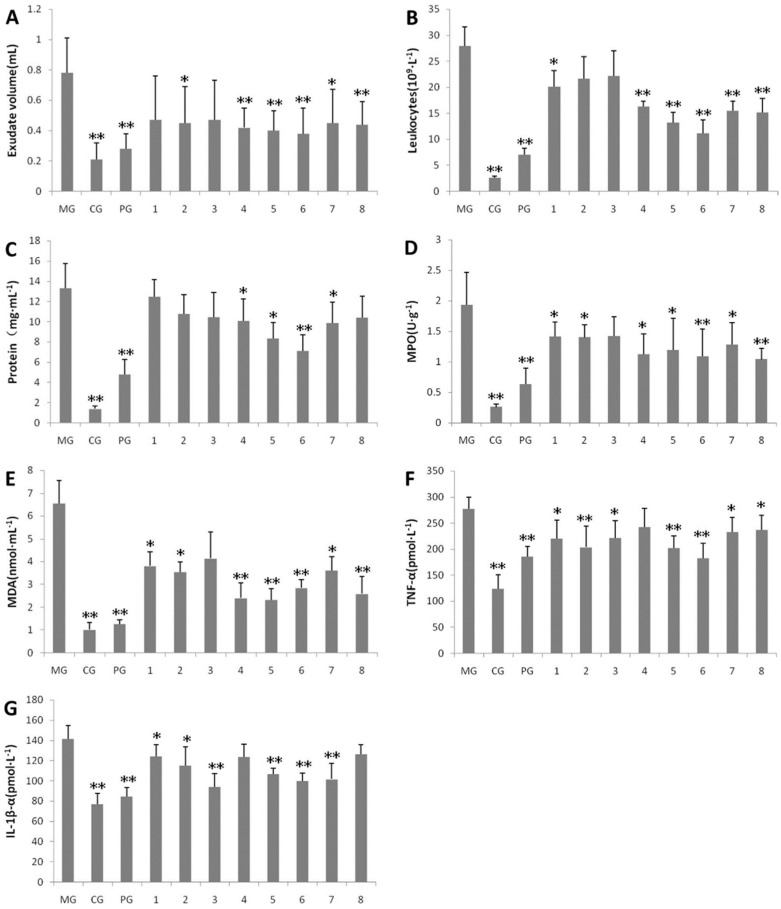
Anti-inflammatory effects of different volatile oils from *ST* on exudate volumes (**A**), leukocytes (**B**), protein levels (**C**), MPO activities (**D**), MDA contents (**E**), TNF-α levels (**F**), IL-1β levels (**G**). Data are expressed as means ± S.E.M.; * *p* < 0.05, ** *p* < 0.01 vs. control group (From Figure 3 in [34]).

The volatile oil, as the main constituent of *ST*, has been widely studied in recent years due to its anti-inflammatory effect. The anti-inflammatory effects of the distilled volatile oils from *ST* on carrageenin-induced pleurisy in rats were investigated by using the *ST* collected at eight different harvesting times. The results demonstrated that the decreases in various indicators such as leukocytes, protein level, myeloperoxidase (MPO), malondialdehyde (MDA), tumor necrosis factor-α (TNF-α) and interleukine-1β (IL-1β) were significant (*p* < 0.01, *p* < 0.05), as depicted in Figure 12 [34].

Wang’s study certified that the anti-inflammatory effects of the extract of *ST* were related to tissue NO and TNF-α suppression, and the decrease in lipid peroxidation and the increase in the activity of antioxidant enzymes including catalase, superoxide dismutase and glutathione peroxidase in vivo [29]. Sohn et al. evaluated the anti-inflammation mechanism of the *ST* extract on the PMA plus A23187-induced stimulation of HMC-1 human mast cells, and they found that *ST* extract can regulate the cytokine–cytokine receptor interaction (CCRI), MAPK and the toll-like receptor (TLR) signaling pathways [35].

Zhang et al. evaluated the effects of the *ST* extracts on 2,4-dinitro-1-chlorobenzene (DNCB)-induced AD skin lesions in Nc/Nga mice, and explored the action mechanism [36]. Miceli et al. studied the aboveground parts of *ST* from a local Greek plant, and found that its methanol extracts such as polyphenols and ursolic acid had a significant inhibitory effect on rat foot edema [37]. The investigation showed that volatile oil from *ST* could significantly reduce the formation of LTB4 and LTC4 as arachidonic acid metabolites [38]. Byun et al. found that the ethanol extracts of *ST* could inhibit the expression of lipopolysaccharide-induced cell surface molecules (CD80 and CD86) and the production of pro-inflammatory cytokines such as TNF-α, IL-1β and IL-6 [39]. Choi et al. found that the extract of *ST* reduced epidermal and dermal thickness in DNCB-induced mice [40]. Moreover, *ST* could inhibit the activity of mitogen-activated protein kinase and the activation of NF-β. Qu’s study showed that *ST* could significantly improve symptoms and pathological tissues of ulcerative colitis model rats and promote intestinal mucosal repair, the mechanism of which may be related to the upregulation of the expression of AQP4 and AQP8 in the colon [41]. In addition, the protective effect of volatile oil extracted from *ST* on endotoxin-poisoned mice was closely related to its anti-inflammatory effect, which was related to the inhibition of NLRP3 inflammasome activation [42].

Kang et al. investigated the anti-inflammatory effects of the aqueous extract of *ST* on LPS-induced TNF-α and IL-6 in vivo [43]. The results suggested that the downregulation of TNF-α by *ST* water extract might inhibit both IkBa degradation and activation of c-Jun and ATF-2.

Bai et al. investigated the spectrum-effect relationship between the GC-MS fingerprint and the antioxidant and anti-inflammatory effects of *ST* essential oil (EO) from various sources, and found that the different sources of *ST* EO exhibited mild antioxidant activities and significant anti-inflammatory effects [44]. Menthone, isomenthone, pulegone, piperitone and β-caryophyllene might be the especially dominant constituents responsible for the antioxidant and anti-inflammatory activities of *ST* EO.

### 3.4. Antitumor Effect

Zang et al. found that the mass concentration of volatile oil of *ST* above 4 mg/mL had a good inhibitory effect on human lung cancer A549 cells [45]. Kim et al. demonstrated that *ST* can inhibit the production of LPS-induced TNF-α and IL-6 [46]. Wu et al. found that *p*-cymene and α-terpinene, as two main components of the volatile oil from *ST*, had a significant lethal effect on MCF-7 cells by blocking the cell cycle, interfering with the antioxidant system of cells, destroying the cell structure and exhibiting significant antitumor activity in vitro [47].

### 3.5. Hemostatic Effect

Jeon et al. found that the *ST* extract effectively inhibited collagen-stimulated platelet function by suppressing MAPK and Akt signaling [48], exhibiting a potential therapeutic effect on the cardiovascular system and platelet function [49]. Zhang et al. found that the *ST* extract could stimulate the in vitro coagulation system of mice, activate the fibrinogen system, shorten the time of tail hemorrhage and liver hemorrhage in mice, and thus, play a hemostatic role [50]. Jeon et al. also believed that the *ST* extract played a hemostatic role by inhibiting the signaling pathway of the mitogen-activated protein kinase (MAPK)/protein kinase B (Akt) [48]. Cao et al. also obtained similar results by performing the experiments on rats using the extract of ethyl acetate [49]. Zhang et al. found that the hemostatic mechanism of *ST* was associated with activating exogenous coagulation pathways and co-coagulation pathways, increasing the number of platelets, improving platelet activation factor TXB2 and reducing the concentration of 6-keto-PGF1α [51].

### 3.6. Abirritation

The volatile oil of the *ST* had a good analgesic effect, which was studied by detecting the changes in the pain threshold of mice before and after the administration of the volatile oil in mouse hot-plate experiments [52]. Huang et al. revealed that the volatile oil of *ST* can increase the pain threshold of mice and significantly improve the cotton ball granuloma of mice through animal experiments, indicating that *ST* had a good anti-inflammatory and analgesic effect [53]. Meng et al. found that the *ST* extract raised the pain threshold of mice and had both peripheral analgesia and central analgesic effects through mouse hot-plate experiments [54].

### 3.7. Antibacterial Effect

Zhu et al. explored the influence of different extraction methods on components and the antibacterial activity of volatile oil from *Forsythiae Fructus*, *Schizonepetae Herba* and *Menthae Haplocalycis Herba*. It was found that the antibacterial effects of volatile oil extracted separately on Escherichia coli, Staphylococcus aureus, Pseudomonas aeruginosa and Candida albicans were better than that and of the physical mixing of volatile oil. [5]. Liang et al. found that the main components of the volatile oil of *ST* from Yunnan wild soil were **α-terpinene**, **terpinol formate** and **p-cymenin**. These compounds had certain inhibitory effects on *Bacillus subtilis*, *Proteus common*, *Escherichia coli*, *Bacillus anthracis*, *Coryneopsis polyspora*, *Fusarium oxysporum* and *Fusarium putrum* [55]. The tests showed that *ST* could eliminate *Helicobacter pylori* and inhibit NF-κB nuclear entry in gastric mucosa to a certain extent [56]. The volatile oil from the leaves and spikes had an antibacterial effect on *B. subtilis*, *S. typhi*, *E. coli* and *S. aureus* [57]. The minimum bactericidal concentration (MBC) of volatile oil in the leaves on *S. typhi* and *E. coli* was 1/6 and 1/24 mg∙mL^−1^, and the MBC of the volatile oil in spikes on *S. typhi* and *E. coli* was 1/12 and 1/24 mg∙mL^−1^. The antibacterial effect of volatile oil in leaves and spikes had a close relationship with the contents of **1-octene-3-ol**, **menthol**, **pulegone** and **caryophyllene oxide**.

### 3.8. Immunomodulatory Effect

The study showed that the treatment with 100 μg/mL of *ST* could suppress the markers of inflammation and allergic reactions, including IL-10, IFN-γ, TNF-*α*, IL-4 and IL-6 [58]. Moreover, 10 μg/mL of *ST* inhibited the release of β-hexosaminidase in RBL-2H3 cells. The results revealed that *ST* had immunomodulatory effects at a cellular level, suggesting the role of *ST* in the treatment of allergic diseases [58]. Oral administration of the *ST* water extract significantly reduced the serum levels of IFN-γ and IL-4 in anti-CD3-treated mice, but enhanced those of IL-2. Similar results were also obtained in anti-CD3-stimulated splenocytes and PBMCs in vitro. Detailed and in-depth study results suggested that the differential regulation of the water extract of *ST* on IFN-γ, IL-4 and IL-2 may be due to its inhibition of NF-kB and enhancement of NFATc2 [59].

Kang et al. investigated the effect of *ST* water extract on the pattern of cytokine production from activated T cells in vivo and in vitro [60]. The mRNA levels of IL-4, IFN-γ and T-BET were significantly reduced after giving *ST* orally (200 mg∙kg^−1^) to mice for 7 days before IV injection of the anti-CD3 antibody. As a Th1-specific transcription factor, T-BET is selectively expressed in Th1 cells, plays an important role in the development of Th1 cells by initiating the Th1 genetic program and inhibits the synthesis of Th2 cytokines. The studies showed that *ST* can regulate inflammation by reducing the release of Th1 and Th2 cytokines from T cells [60]. Kim et al. studied the anti-inflammatory mechanism of *ST* in mouse peritoneal macrophages, and revealed that *ST* inhibited LPS-induced TNF-*α* and IL-6 production [60]. The maximal inhibition rate of *ST* (2 mg/mL) on the production of TNF-*α* and IL-6 was 48.01 ± 2.8% and 56.45 ± 2.8%, respectively [46].

Zhu et al. extracted the polysaccharide from *ST* spikes by water extraction and the alcohol precipitation method, and determined the total sugar content of the polysaccharide by the phenol-sulfuric acid method [61]. The results showed that 200, 400 and 800 μg·mL^−1^ of HSP can promote the proliferation of macrophages, enhance the phagocytic activity of macrophages, improve the spleen index and thymus index of immunosuppressed mice, and have a certain regulatory effect on the immune system. Yang et al. administered an *ST* decoction to a cough model in mice and found that lymphocyte subsets (CD4+, CD8+) in spleen and immune factor (IL-1, IL-6) levels in serum were significantly increased, indicating that the *ST* decoction could regulate immune function indexes of mice [62]. Fan et al. applied an *ST* forsythia decoction to a chronic eczema model in mice, and detected the spleen index of immune organs and the ratio of CD4+/CD8+ lymphocytes in the spleen [63]. The results showed that the *ST* forsythia decoction group could significantly inhibit the increase in the spleen index and the ratio of CD4+/CD8+ cells. The experimental results showed that the *ST* forsythia decoction had the effect of immune regulation. In conclusion, *ST* plays a protective role in SV40 MES13 cells by reducing MG-induced cytotoxicity and oxidative stress. It could also prevent the formation of MG-mediated AGEs and inhibit AGE-protein crosslinking. It can be surmised that *ST* can be an effective therapeutic approach, as a functional food, for diabetes [63].

The effects of the water extract of *ST* on immediate allergic reactions were studied. The results showed that the water extract of *ST* could weaken the histamine release from human mast cell line (HMC-1) cells, and inhibit the immunoglobulin E (IgE)-mediated skin allergic reaction and compound the 48/80-induced systemic allergic reaction [64].

### 3.9. Antiviral Activity

**Figure 13:**
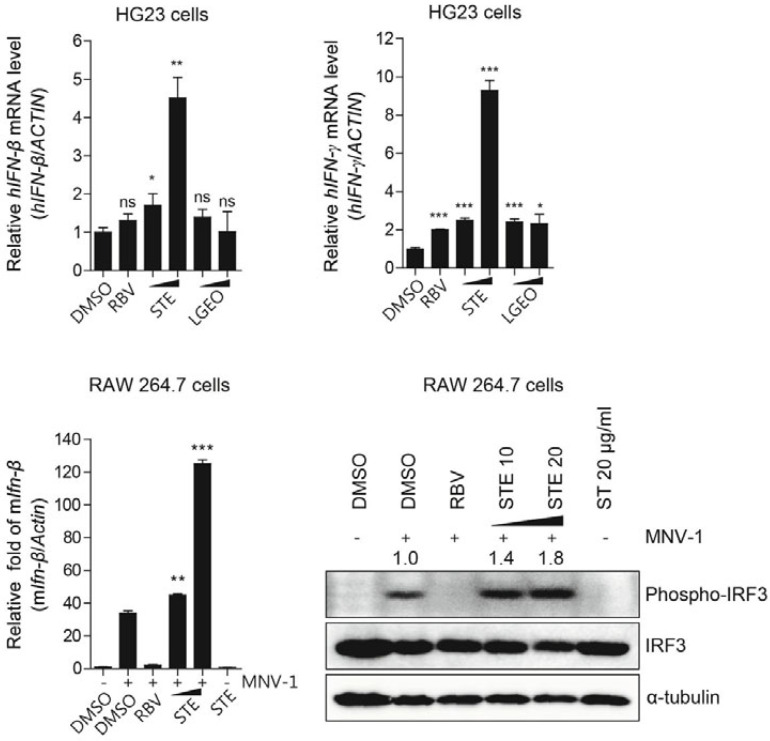
Induction of antiviral interferon production by STE during norovirus replication (* *p* < 0.05, ** *p* < 0.01, *** *p* < 0.001 versus control (*t*-test)) [65]. (Detailed information can be found in Figure 4 of [65]).

Ng et al. studied the antiviral activity of the *ST* extract against norovirus [65]. Human norovirus replicon-bearing HG23 cells were treated with the *ST* extract at 5 and 10 mg/mL, and the viral RNA levels were reduced by 77.2% and 85.9%, respectively. They examined the effect of STE on innate immunity during norovirus replication (Figure 13). The treatment of the *ST* extract induced the expression of mRNAs for type I and type II interferons in HG23 cells and upregulated the transcription of interferon-β in infected RAW 264.7 cells via increased phosphorylation of interferon regulatory factor 3, a critical transcription regulator for type I interferon production. Taken together, these results suggested that *ST* extract can inhibit human and mouse norovirus replication by inducing antiviral IFN production during viral replication, and *ST* extract may serve as a candidate antiviral substance for the treatment of norovirus [65]. Chen et al. reported that the aqueous extract of *ST* could inhibit the replication of enterovirus 71 (EV71), suggesting that the water extract of *ST* had anti-EV71 activity and can be used as a health food or a candidate drug for EV71 prevention [66].

### 3.10. Other Pharmacological Effects

Kim et al. examined the effects and mechanisms of the action of the ethanolic extract of *ST* on osteoclastogenesis in vitro in bone marrow macrophages (BMMs) stimulated with the receptor activator of the nuclear factor kappa-B ligand (RANKL) and in vivo using a mouse model of LPS-induced bone destruction [67]. They found that the ethanolic extract of *ST* was a potential agent for the treatment of osteoclast-related bone diseases, such as osteoporosis [67].

In addition, Yang et al. evaluated the acaricidal potential of **2-isopropyl-5-methyl-cyclohexanone** isolated from *ST* oil and its structurally related derivatives [68]. Their results indicate that the *ST* oil and the **2-isopropyl-5-methylcyclohexanone** structural analogues may be potential agents for controlling indoor dust and stored food mites, and may protect humans from indoor allergens [68].

## 4. Conclusions

In summary, the main chemical constituents of *ST* include monoterpenoids, sesquiterpenoids, flavonoids, aldehydes, ketones, quinones, alcohols, phenols, carboxylic acids, esters, alkenes and alkanes. According to the literature, it was found that the types and contents of chemical constituents obtained from different medicinal parts of *ST* or the same part of *ST* obtained through different extraction methods are different. The pharmacological effects are mainly reflected as antipyretic effects, antioxidant effects, hypolipemic effects, anti-inflammatory effects, immunomodulatory effects, hemostatic effects, abirritation, antitumor effects, antibacterial effects and antiviral activity. The mechanisms of abirritation, bacteriostasis, antiviral activity, etc. are not systematic and complete, and regulating the signaling pathway to exert pharmacological effects needs further study. In addition, the pharmacological effects of water or alcohol extracts of *ST* were studied more than the specific chemical constituents of *ST*. Therefore, with the development of science and technology, more chemical constituents will be isolated and their pharmacological effects will be better understood.

## Figures and Tables

**Table 1:** Quantitative analysis of volatile oil of *ST* and *MH* [5].

Entry	Constituents	CAS	*ST*/%	*MH/*%
1	1-Octen-3-ol	003391-86-4	0.73	-
2	Limonene	005989-27-5	5.00	1.80
3	1-octen-3-ol	002442-10-6	0.44	-
4	Iso-menthone	000491-07-6	43.58	-
5	Pyridazine	000089-80-5	2.71	11.90
6	Trans-isopulegone	029606-79-9	1.43	-
7	Pulegone	000089-82-7	40.02	14.52
8	Piperitone	000491-09-8	1.12	1.32
9	Caryophyllene oxide	001139-30-6	1.38	1.31

**Table 2:** The contents of eight components in *ST* from 13 different origins in China (mg/g) [21].

Entry	Caffeic Acid	Cynaroside	Quercitrin	Rosmarinic Acid	Luteolin	Apigenin	Diosmetin	Pulegone
S1	0.0691	0.1168	0.6116	1.5821	0.3994	0.0436	0.0839	1.4944
S2	0.0341	0.0925	0.2653	0.9058	0.1725	0.0227	0.0472	0.7942
S3	0.0333	0.1897	0.0372	0.3063	0.2925	0.0234	0.0657	0.2097
S4	0.0366	0.0968	0.6996	1.2986	0.3824	0.0467	0.1074	0.8702
S5	0.0597	0.1228	0.5311	1.2404	0.7753	0.0312	0.0681	1.1241
S6	0.1049	0.0734	0.5406	0.8233	0.3251	0.0428	0.0999	1.0054
S7	0.0846	0.1242	0.7346	1.3433	0.5298	0.0602	0.0741	1.6335
S8	0.1697	0.1830	0.6159	1.5052	0.4300	0.0630	0.0571	12.4694
S9	0.2020	0.1070	0.4136	2.0761	0.3759	0.1009	0.0392	20.7663
S1	0.1637	0.1487	1.0849	2.5900	0.5221	0.0731	0.0885	35.9777
S1	0.0638	0.0848	0.8173	1.4188	0.2397	0.0476	0.0991	0.8803
S1	0.0847	0.0554	0.3971	0.8774	0.5181	0.0554	0.0533	0.8091
S1	0.2359	0.1315	0.5864	2.0414	0.1905	0.1104	0.0353	9.2541

## Data Availability

Not applicable.

## References

[1] Pharmacopoeia Commission of the Ministry of Public Health (2020). Chinese Pharmacopoeia.

[2] Chun M.H., Kim E.K., Yu S.M., Oh S.M., Moon K.Y., Jung J., Hong J. (2011). GC/MS combined with chemometrics methods for quality control of *Schizonepeta tenuifolia* Briq.: Determination of essential oils. Microchem. J..

[3] Yu S., Chen Y.W., Zhang L., Shan M.Q., Tang Y.P., Ding A.W. (2011). Quantitative Comparative Analysis of the Bio-Active and Toxic Constituents of Leaves and Spikes of *Schizonepeta tenuifolia* at Different Harvesting Times. Int. J. Mol. Sci..

[4] Chun M.H., Kim E.K., Lee K.R., Jung J.H., Hong J.K. (2010). Quality control of *Schizonepeta tenuifolia* Briq. by solid-phase microextraction gas chromatography/mass spectrometry and principal component analysis. Microchem. J..

[5] Zhu M.F., Tang Y., Zheng Q., Tang D.F., Luo J., Hu P.Y., Guo Y.Y., Wu H.X., Yang M. (2018). Effects of different extraction methods on composition and antibacterial activity of volatile oil from *Forsythiae Fructus*, *Schinzonepetae Herba*, and *Menthae Haplocalycis Herba*. Chin. Tradit. Herb. Drugs..

[6] Cai S.F., Chen K.H., Linghu R.G. (2007). Supercritical CO_2_ Extraction of Volatile Oil from *Schizonepeta tenuifolia* Briq. and GC-MS Analysis. J. Nanjing For. Univ. Nat. Sci. Ed..

[7] Yu P., Qiu Q., Cui Z.J., Liu S., Liu T.L. (2002). Determination of chemical constituents of the essential oil from *Schizonepeta tenuifolia* Briq. by GC-MS. Chin. Tradit. Pat. Med..

[8] Liu X.Q., Li S.W., Li Z.M., Zhang X.D., Cui X.Z., Lu C.M. (2008). Study on the constituents of volatile oil from different parts of *Schizonepeta tenuifolia* Briq. Chin. Tradit. Herb. Drugs.

[9] Qiu Q., Ling J.Y., Ding Y.P., Chang H.W., Wang J., Liu T.L. (2005). Comparison of Supercritical Fluid Extraction and Steam Distillation Methods for the Extraction of Essential Oils from *Schizonepeta tenuifolia* Briq. Chin. J. Chromatogr..

[10] Chen Y., Jiang Z.H., Tian J.K. (2006). GC-MS Analysis of Volatile Oil from the Ear of *Schizonepeta tenuifolia* Briq. J. Chin. Med. Mater..

[11] Du C.Z., Qin J.P., Chen Y.P., Cai Y. (2014). GC-MS Analysis of Volatile Oil Components in *Schizonepeta tenuifolia* Briq. from Various Habitats. Hubei Agric. Sci..

[12] Ye D.J., Ding A.W., Yu L., Cao G.X., Wu M. (1985). Study on the components of volatile oil from different medicinal parts and after carbon frying of *Schizonepeta tenuifolia* Briq. Chin. J. Chin. Mater. Med..

[13] Wu Y.L., Ding A.W., Feng Y.L. (2000). Study on volatile oil of *Schizonepeta tenuifolia* Briq. and its related Medicinal materials. Chin. Tradit. Herb. Drugs.

[14] Yang Z.Y., Yan J.C., Zhang S.J., Yu L.G. (1996). Study on chemical constituents of volatile oil from *Schizonepeta Spica*. Chin. Tradit. Herb. Drugs..

[15] Meng N., Huang S., Hu D.D., Xu Y.L., Wang F.Y., Wang J.L. (2017). Chemical constituents from *Nepeta angustifolia*. Chin. Tradit. Pat. Med..

[16] Lee I.K., Kim M.A., Lee S.Y., Hong J.K., Lee J.H., Lee K.R. (2008). Phytochemical Constituents of *Schizonepeta tenuifolia* Briquet. Nat. Prod. Sci..

[17] Zhao L.Z., Jin Y., Han X.M., Chu Q.L., Yang J.S. (2017). Two new sesquiterpenes from the aerial parts of *Schizonepeta tenuifolia*. J. Asian Nat. Prod. Res..

[18] Zeren L.M., Pu Z., Zhuoma D.Z., Wang J.L. (2014). Chemical constituents and pharmacological action of *Schizonepeta tenuifolia* Briq. J. Mod. Med. Health.

[19] Fang J.X., Wang S., Meng X.S., Bao Y.R., Li T.J. (2017). Determination of six flavonoids in *Schizonepeta tenuifolia* from different areas by HPLC. Chin. Tradit. Herb. Drugs..

[20] Song K., Wang H.Q., Liu C., Kang J., Li B.M., Chen R.Y. (2014). Chemical Studies on the Herb of *Chenopodium ambrosioides*. Chin. J. Chin. Mater. Med..

[21] Wen Z.S., Liu L.L., Li X.R., Zheng Y.G., Ma D.L. (2019). Simultaneous determination of 8 components in *Schizonepeta tenuifolia* from different habitats by HPLC. J. Chin. Med. Mater..

[22] Huang X.H., Chen J., Xu X.Q., Zhang W.T., Zhao C.C. (2016). A New Phenolic Compound from *Schizonepeta tenuifolia*. Chem. Nat. Compd..

[23] Zhang L., Zhang M., Sun E., Ding A.W. (2008). Anit-inflammatory, analgesic and antipyretic effects of *schizonepetolide* poly-lactic-CO-glycolic acid nanoparticles. J. China Pharm. Univ..

[24] Cai G.X., Liu L., Wang Y.H., Tang Z.P. (2008). Comparison of powders with different diameters of *schizonepeta* on anti-pyretic and analgesic effects. J. Hunan Univ. Chin. Med..

[25] Song L. (2007). Pharmaceutical Study of JG Spray. Ph.D. Thesis.

[26] Wu Z.Q., Liu G.H., Yan L.J., Nan C.H., Yue Z.J., Wang X.F. (2010). Experimental study on anti-influenza virus infection with yinqiao-decoction by orthogonal design. Chin. J. Exp. Clin. Virol..

[27] Bouididael E.H., Alaoui K., Cherrah Y., Chammache M., Idrissi A.I. (2008). Analgesic activity of different nonvolatile extracts of *Nepeta atlantica* Ball and *Nepeta Tuberosa* L. ssp. reticulata (Desf.) Maire. Therapie.

[28] Tae K.S., Kim S.J. (2012). Inhibition of iNOS and DNA Oxidation by Methanol Extract of *Schizonepeta tenuifolia*. Trop. J. Pharm. Res..

[29] Wang B.S., Huang G.J., Tai H.M., Huang M.H. (2012). Antioxidant and anti-inflammatory activities of aqueous extracts of *Schizonepeta tenuifolia* Briq. Food Chem. Toxicol..

[30] Wen Z.S., Li X.R., Fan Z.X., Ma D.L., Zheng Y.G. (2019). Study on antioxidant activity of *Schizonepeta tenuifolia* polysaccharide extract. Hebei J. Tradit. Chin. Med..

[31] Do M.H., Choi J., Kim Y., Park H.Y., Ha S.K., Hur J. (2019). *Schizonepeta tenuifolia* reduces methylglyoxal-induced cytotoxicity and oxidative stress in mesangial cells. J. Funct. Foods..

[32] Berner A.K., Brouwers O., Pringle R., Klaassen I., Colhoun L., McVicar C., Brockbank S., Curry J.W., Miyata T., Brownlee M. (2012). Protection against methylglyoxal-derived AGEs by regulation of glyoxalase 1 prevents retinal neuroglial and vasodegenerative pathology. Diabetologia.

[33] Qian R.H., Ren Q.R., Huang J., Zhou Y.J., Zhang J.J., Wang Y.N. (2018). Study on the ldentification and Antioxidant and Antibacterial Activity of Total Flavonoids from Chenopodium ambrosioides L. in Sichuan Province. Food Ind..

[34] Shan M.Q., Qian Y., Yu S., Guo S.C., Zhang L., Ding A.W., Wu Q.N. (2016). Anti-inflammatory effect of volatile oil from *Schizonepeta tenuifolia* on carrageenin-induced pleurisy in rats and its application to study of appropriate harvesting time coupled with multi-attribute comprehensive index method. J. Ethnopharmacol..

[35] Sohn S.H., Cho S., Ji E.S., Kim S.H., Shin M., Hong M., Bae H. (2012). Microarray analysis of the gene expression profile of HMC-1 mast cells following *Schizonepeta tenuifolia* Briquet treatment. Cell Immunol..

[36] Zhang T., Qiu J., Wu X., Huang S., Yuan H., Park S. (2020). *Schizonepeta Tenuifolia* with Alpinia Oxyphylla Alleviates Atopic Dermatitis and Improves the Gut Microbiome in Nc/Nga Mice. Pharmaceutics.

[37] Miceli N., Taviano M.F., Giuffrida D., Trovato A., Tzakou O., Galati E.M. (2005). Anti-inflammatory activity of extract and fractions from Nepeta sibthorpii Bentham. J. Ethnopharmacol..

[38] Zeng N., Shen Y.J., Ren Y.X., Li J.H. (2006). Experimental study on anti-inflammatory mechanism of volatile Oil from *Schizonepeta tenuifolia*. J. Chin. Med. Mater..

[39] Byun M.W. (2014). *Schizonepeta tenuifolia* ethanol extract exerts anti-inflammatory activity through the inhibition of TLR4 signaling in lipopolysaccharide-stimulated macrophage cells. J. Med. Food.

[40] Choi Y.Y., Kim M.H., Kim J.H., Jung H.S., Sohn Y., Choi Y.J., Hwang M.K., Kim S.H., Kim J., Yang W.M. (2013). *Schizonepeta tenuifolia* inhibits the development of atopic dermatitis in mice. Phytother. Res..

[41] Qu Y., Zhang S.X., Fu L.Y., Dai Q.Y., Zhang Y.B., Liu Z.H., Li S.Y., Yang X.Y., Nie G.K., Wang R. (2020). Effect of *Schizonepetae Herba* and *Saposhnikoviae Radix* on expression of AQP4 and AQP8 in colonic mucosa of rats with ulcerative colitis. Chin. J. Chin. Mater. Med..

[42] Lv H.J., Wen T.Q., Luo J., Liu X.B., Yang J., Zeng N. (2019). Study on mechanism of essential oils of *Schizonepeta tenuifolia* Briq. in endotoxin poisoning mice via NLRP3 inflammasome pathway. Chin. Pharmacol. Bull..

[43] Kang H., Han S.W., Hong J.W., Sohn N.W. (2010). Suppression of tumour necrosis factor-a by *Schizonepeta tenuifolia* water extract via inhibition of IkBa degradation and Jun N-terminal kinase/stress-activated protein kinase activation. J. Pharm. Pharmacol..

[44] Bai X., Liu L., Zhang J.P., Chen L., Wu T., Aisa H.A., Maiwulanjiang M. (2021). Spectrum–effect relationship between GC-QTOF-MS fingerprint and antioxidant, anti-inflammatory activities of *Schizonepeta tenuifolia* essential oil. Biomed. Chromatogr..

[45] Zang L.Q., Hu F., Wei M., Wang N.P., Wei J.B. (2006). Research on Anti-tumor Effect of Essential Oil in *Schizonepeta Tenuifolia* Briq. and Its Inducing Apoptosis. Guangxi J. Tradit. Chin. Med..

[46] Kim S.J., Kim J.S., Choi I.Y., Kim D.H., Kim M.C., An H.J., Na H.J., Kim N.H., Moon P.D., Myung N.Y. (2008). Anti-Inflammatory Activity of *Schizonepeta tenuifolia* through the Inhibition of MAPK Phosphorylation in Mouse Peritoneal Macrophages. Am. J. Chin. Med..

[47] Wu J.L. (2014). Antitumor Mechanism of Essential Oil and Its Two Main Components from *Chenopodium ambrosioides* L. on Human Breast Cancer MCF-7 Cells. Doctoral Dissertation.

[48] Jeon B.R., Irfan M., Kim M., Lee S.E., Lee J.H., Man H.R. (2019). *Schizonepeta tenuifolia* inhibits collagen stimulated platelet function via suppressing MAPK and Akt signaling. J. Biomed. Res..

[49] Cao L.L., Li X., Zhang L. (2010). Experimental study on the effect of *Schizonepeta Spica Carbonisata* and its effective parts on rat coagulation system. Chin. Tradit. Pat. Med..

[50] Zhang M.L., Zhao Y., Cheng J.J., Liu X.M., Wang Y.Z., Yan X., Zhang Y., Lu F., Wang Q.G., Qu H.H. (2018). Novel carbon dots derived from *Schizonepetae Herba* Carbonisata and investigation of their haemostatic efficacy. Artif. Cells. Nanomed. Biotechnol..

[51] Zhang M.L. (2018). Study on Hemostatic Substance Basis and Mechanism of Charred Schizonepeta.

[52] Xue Z.B. (2014). Preparation, Quality Analysis and Activity Evaluation of a Traditional Chinese Medicated Oil with Anti-Inflammatory and Analgesic Effect.

[53] Huang S., Jiang C.Y., Long F. (2011). Anti-Inflammation and Analgesic Effect of Volatile Oil from *Nepeta Angustifolia*. Her. Med..

[54] Meng N., Huang S., Hu D.D., Yuan R.Y., Li B., Wang J.L., Wu C.Y. (2018). Anti-Inflammatory and Analgesic Effects of Different Solvent Extractions of *Nepeta Angustifolia*. Chin. Arch. Tradit. Chin. Med..

[55] Liang Q., Liu W.Y., Zhang Q., Wang F. (2018). Chemical constituents and anti-microbial activities of essential oil of *Chenopodium ambrosioides L.* from Kunming Yunnan. Lishizhen Med. Mater. Med. Res..

[56] Ye H., Yu J., Zhang X.Z. (2017). Study on bactericidal activity against H.pylori in vivo and effects on the NF-κB expression of *Chenopodium ambrosioides* L. volatile oil. Chin. J. Tradit. Chin. Med..

[57] Su C., Zhao Y.Y., Feng Y., Zhang L.L., Zheng Y.G., Zheng K.Y. (2022). Antibacterial effect and composition analysis of the leaves and spicas from *Schizonepeta tenuifolia* Briq. Chin. J. New Drugs.

[58] Lin Y.H., Chen H.Y., Chiu J.C., Chen K.J., Ho H.Y., Yang S.H. (2018). Immunomodulation Effects of *Schizonepeta tenuifolia* Briq. On the IgE-Induced Allergic Model of RBL-2H3 Cells. Evid.-Based Complementary Altern. Med..

[59] Kang H., Moon J.Y., Sohn N.W. (2010). Regulation of interferon-gamma, interleukin-4 and interleukin-2 by *Schizonepeta tenuifolia* through differential effects on nuclear factor-kappaB, NFATc2 and STAT4/6. Exp. Biol. Med..

[60] Kang H., Oh Y.J., Choi H.Y., Ham I.H., Bae H.S., Kim S.H., Ahn K.S. (2008). Immunomodulatory effect of *Schizonepeta tenuifolia* water extract on mouse Th1/Th2 cytokine production in-vivo and in-vitro. J. Pharm. Pharmacol..

[61] Zhu Y.D., Li Z.K., Liang Y.Q., Xia H.Y., Li C.H., Wang C.J. (2021). Immunomodulatory Activity of Polysaccharides from *Herba Schizonepetae*. Her. Med..

[62] Yang M.H., Huang X.W., Xin G., Liu Y.X., Zhao X.T., Li X., Xu Y., Zhang Q. (2019). Experimental study on the best decoction time of *Schizonepeta* in *Schizonepeta decoction*. Chin. J. Hosp. Pharm..

[63] Fan H.J., Lu Z.W., Wen F., Xie Z.P., Xie J.W., Pei T.T., Tan Z.B., Wu Y.T., Liu B., Zhou Y.C. (2018). Anti-inflammation and Restoring Immune Function Effects of Jingjie Lianqiao Decoction in Chronic Atopic Dermatitis Mice. Liaoning J. Tradit. Chin. Med..

[64] Yoo J.S., Kim D.K., Kim S.H., Shin T.Y. (2011). Anti-allergic Effects of *Schizonepeta tenuifolia* on Mast Cell-Mediated Allergy Model. Nat. Prod. Sci..

[65] Ng Y.C., Kim Y.W., Lee J.S., Lee S.J., Song M.J. (2018). Antiviral activity of *Schizonepeta tenuifolia* Briquet against noroviruses via induction of antiviral interferons. J. Microbiol..

[66] Chen S.G., Cheng M.L., Chen K.H., Horng J.T., Liu C.C., Wang S.M., Sakurai H., Leu Y.L., Wang S.D., Ho H.Y. (2017). Antiviral activities of *Schizonepeta tenuifolia* Briq. against enterovirus 71 in vitro and in vivo. Sci. Rep..

[67] Kim J.Y., Baek J.M., Ahn S.J., Cheon Y.H., Park S.H., Yang M., Choi M.K., Oh J. (2016). Ethanolic extract of Schizonepeta tenuifolia attenuates osteoclast formation and activation in vitro and protects against lipopolysaccharide-induced bone loss in vivo. BMC Complementary Altern. Med..

[68] Yang J.Y., Hoi-Seon Lee H.S. (2013). Changes in Acaricidal Potency by Introducing Functional Radicals and an Acaricidal Constituent Isolated from *Schizonepeta tenuifolia*. J. Agric. Food Chem..

